# Sequencing the B Cell Receptor Repertoires of Antibody-Deficient Individuals With and Without Infection Susceptibility

**DOI:** 10.1007/s10875-023-01448-0

**Published:** 2023-02-24

**Authors:** Yoong Wearn Lim, Neftali Jose Ramirez, Michael A. Asensio, Yao Chiang, Gabriele Müller, Pavla Mrovecova, Noriko Mitsuiki, Máté Krausz, Nadezhda Camacho-Ordonez, Klaus Warnatz, Adam S. Adler, Bodo Grimbacher

**Affiliations:** 1 GigaGen, Inc. (A Grifols Company), San Carlos, CA USA; 2 Institute for Immunodeficiency, Medical Center, Faculty of Medicine, Albert-Ludwigs University, Freiburg, Germany; 3 Center for Chronic Immunodeficiency (CCI), Medical Center, Faculty of Medicine, Albert-Ludwigs University, Freiburg, Germany; 4 Department of Pediatrics and Developmental Biology, Graduate School of Medical Sciences, Tokyo Medical and Dental University, Tokyo, Japan; 5 Department of Rheumatology and Clinical Immunology, Medical Center, Faculty of Medicine, Albert-Ludwigs University, Freiburg, Germany; 6 Faculty of Biology, Albert-Ludwigs University, Freiburg, Germany; 7 DZIF – German Center for Infection Research, Satellite Center Freiburg, Freiburg im Breisgau, Germany; 8 CIBSS – Centre for Integrative Biological Signalling Studies, Albert-Ludwigs University, Freiburg, Germany; 9 RESIST – Cluster of Excellence 2155 to Hanover Medical School, Satellite Center, Freiburg, Germany

**Keywords:** Antibody repertoire sequencing, hypogammaglobulinemia, infection susceptibility

## Abstract

**Purpose:**

Most individuals with antibody deficiency (hypogammaglobulinemia) need immunoglobulin replacement therapy (IgG-RT) from healthy plasma donors to stay clear of infections. However, a small subset of hypogammaglobulinemic patients do not require this substitution therapy. We set out to investigate this clinical conundrum by asking whether the peripheral B cell receptor repertoires differ between antibody-deficient patients who do and do not need IgG-RT.

**Methods:**

We sequenced and analyzed IgG and IgM heavy chain B cell receptor repertoires from peripheral blood mononuclear cells (PBMCs) isolated from patients with low serum IgG concentrations who did or did not require IgG-RT.

**Results:**

Compared to the patients who did not need IgG-RT, those who needed IgG-RT had higher numbers of IgG antibody clones, higher IgM diversity, and less oligoclonal IgG and IgM repertoires. The patient cohorts had different heavy chain variable gene usage, and the patients who needed IgG-RT had elevated frequencies of IgG clones with higher germline identity (i.e., fewer somatic hypermutations).

**Conclusion:**

Antibody-deficient patients with infection susceptibility who needed IgG-RT had more diverse peripheral antibody repertoires that were less diverged from germline and thus may not be as optimal for targeting pathogens, possibly contributing to infection susceptibility.

**Supplementary Information:**

The online version contains supplementary material available at 10.1007/s10875-023-01448-0.

## Introduction

Defense against infections is orchestrated by a complex immune system where every component has a task, and the quantitative or qualitative defect of a single component often contributes to a clinically apparent immunodeficiency [1]. The most common form of inborn errors of immunity/primary immunodeficiency is antibody deficiencies, a phenotype which is mostly characterized by recurrent upper respiratory tract infections. Antibody deficiencies include agammaglobulinemia (no antibodies), hypogammaglobulinemia (not enough antibodies), IgG subclass deficiencies, and specific anti-PnPS (pneumococcal polysaccharide) deficiency, the latter presenting with recurrent pneumococcal infections [2]. The combination of serum IgG levels and infections’ susceptibility are used to make the decision for or against providing IgG-RT, as the immunoglobulin replacement preparations do not contain significant amounts of IgM or IgA. Hence, IgG-RT is not indicated for the treatment of selective IgA deficiency [3]. The reduction of an IgG titer to 4 g/L has been shown to be associated with an increased risk of infection [4], though some patients with almost normal IgG levels may still present with pathological infection susceptibility. Conversely, some people with IgG levels of <4 g/L show no apparent infection susceptibility, potentially because their immune system can respond to each challenge with high-quality acute naïve and memory IgG responses [5].

We approached this clinical conundrum with the question whether the composition of the peripheral B cell receptor sequences and number/diversity of B cell clones may provide an indication of why some patients with severe hypogammaglobulinemia have no infection susceptibility and thus do not need IgG-RT, while most of the patients with antibody deficiency need IgG-RT to stay healthy. We sequenced and analyzed the IgG and IgM heavy chain B cell receptor repertoires from PBMCs isolated from cohorts of patients with low serum IgG concentrations who did or did not require IgG-RT. We found that patients who needed IgG-RT had more diverse IgG and IgM antibody repertoires, and their IgG sequences were significantly more similar to germline. This suggests that, although patients with low serum IgG concentrations who required IgG-RT had higher diversity repertoires, their antibody clones were less diverged from germline and thus might not be as optimal for targeting pathogens, causing infection susceptibility. Conversely, those with low serum IgG concentrations who did not need IgG-RT had less diverse, yet more matured, antibody sequences, which might be better suited to targeting pathogens. The identification of the latter sequences may lead to the production of synthetic immunoglobulin molecules well suited to protect recipients from infections.

## Methods

### Sample Collection

We identified patients from the adult outpatient immunodeficiency clinic of the University of Freiburg with decreased levels of serum IgG (<4 g/L) and remaining peripheral B cells of >40/μL. In the case of patients with the need for IgG-RT (those who had recurrent infections of the respiratory tract, *n* = 15), hypogammaglobulinemia was evaluated using retrospective data from the time of diagnosis (before starting regular IgG-RT). Hypogammaglobulinemia patients that did not have recurrent respiratory tract infections (*n* = 10) were not prescribed IgG-RT. The patient’s infection history, other non-infectious diagnoses, and their ability to respond to vaccines are provided in Table S1.

The participating individuals donated blood samples after signing an informed written consent. PBMCs from the donated blood samples were isolated using Ficoll/Pancoll density gradient centrifugation under sterile conditions, following standard protocols. The harvested PBMCs (9–17 × 10^6^ cells/mL) in freezing medium (heat-inactivated 90% fetal bovine serum (FBS) + 10% dimethyl sulfoxide (DMSO)) were stored in liquid nitrogen until further processing.

### Flow Cytometry

Red blood cells from 500 μL whole blood were lysed for 10 min at 4°C with ammonium chloride, washed twice with phosphate-buffered saline (PBS) + 2% FBS, and stained with anti-CD19 (APC-Cy7, HIB19, Biolegend), anti-CD27 (BV421, M-T271, Biolegend), anti-IgD (PE, IA6-2, Biolegend), anti-IgA (FITC, goat IgG, Southern Biotech), and anti-IgG (AF700, G18-145, BD Biosciences) for 20 min at room temperature. Subsequent fixation (Optilyse B, Beckman Coulter) for 20 min at room temperature was followed by another washing step with PBS + 2% FBS. Stained cells were measured with a Navios Flow Cytometer (Beckman-Coulter) and analyzed with Kaluza Analysis Software (Beckman-Coulter).

### Antibody Repertoire Sequencing

The harvested PBMCs were thawed into media (RPMI + 10% FBS) and counted on a Cellometer K2 (Nexcelom). The cells were pelleted by centrifugation and RNA was extracted using a NucleoSpin RNA Plus kit (Macherey-Nagel) according to manufacturer’s instructions. To amplify heavy chain variable regions for deep sequencing, tailed-end RT-PCR was performed on the extracted RNA. At the 5’ end, a pool of variable region primers with Illumina adapters was used, and at the 3’ end, a constant region primer (for IgG or IgM) with a sample-specific index sequence and Illumina adapter was used (Table S2); IgG and IgM sequences were amplified in separate reactions [6]. The PCR product was run on an agarose gel, extracted, purified, and quantified using a KAPA quantitative PCR Illumina Library Quantification Kit (1069, Roche). The libraries were sequenced as previously described [7] on a MiSeq (Illumina) at a library concentration of 9 pM with a 255-cycle forward read and a 255-cycle reverse read (see Table S2 for sequencing primers). Sequencing data are available in the Short Read Archive under project identifier PRJNA876301.

### Antibody Sequence Analysis

We sequenced the antibody repertoire libraries to an average of 28,901 reads (range 13,064–45,080 reads). Sequence analysis was performed using our previously reported bioinformatics pipeline [7–9]. Briefly, we calculated the expected number of errors (E) for a read from its Phred scores and discarded reads with E >2 [10]. After error filtering, we randomly sampled up to 15,000 reads from each sample for further analysis. We verified that our findings were consistent across multiple rounds of random read sampling (data not shown). We processed IMGT [11] immunoglobulin sequences to generate position-specific sequence matrices (PSSMs) for each framework/CDR junction. We used these PSSMs to identify framework/CDR junctions for each of the nucleotide sequences. Python scripts were then used to translate the sequences. We required reads to have a valid predicted CDR3 sequence. We then defined antibody “clones” conservatively, where unique sequences were combined if they had 1 amino acid difference for 5–6 amino acid long CDR3H, or if they had 1–2 amino acid differences for >6 amino acid long CDR3H. Only clones with at least two sequencing reads were included in the analysis.

We ran UBLAST [12] using the nucleotide sequences as queries and V and J gene sequences from the IMGT database as the reference sequences. The UBLAST alignment with the lowest E-value was used to assign V and J gene families and compute percent identity to germline [7–9]. The IgG sample for patient CVID-1712-01 had low sequence quality and was excluded from analysis.

### Antibody Diversity Index

Antibody diversity index was calculated using the *diversity* function of the *tcR* package (version 2.3.2) [13] in R version 4.1.2.

### Correlation Analysis

The data used for the correlation analysis are in Table S1. Pearson correlation analysis was performed using the *cor* function of the *corrplot* package (version 0.92) [14] using the “pairwise.complete.obs” option, in R version 4.1.2. Correlations with *P* ≤ 0.05 were considered significant.

### Variable Gene Usage and Mutation Frequency

To identify IGH V gene identity, sequencing fasta files were mapped to human V gene reference sequences (release 202243-1, 24 October 2022) from IMGT [11], using *USEARCH* version v8.1.1916M_i86linux64 (options: -usearch_local -mismatch -1 -id 0.5 -evalue 1e-3) [12]. The IMGT antibody numbering system was used to identify CDR and framework regions along V genes (which was also used to determine CDR3H length). For the principal component analysis (PCA), we added a pseudo count of 1 to all V gene frequencies and log2 transformed them. PCA was performed using the *prcomp* command in R. Wilcoxon rank sum tests were used to compare V gene usage frequencies between donors who did and did not need IgG-RT. *P*-values were adjusted for the number of V genes tested using the Benjamini-Hochberg method. The number of mismatches along V genes was tallied using custom Perl scripts and visualized using *ggplot2* [15] in R. The first 21 nucleotides (7 amino acids) of V genes were the PCR primer binding sites for preparing the antibody sequencing libraries. Any mutations in this region could not be accurately measured and thus the region was excluded from the V gene mutation frequency analysis.

## Results

### Patient Cohorts

**Fig. 1:**
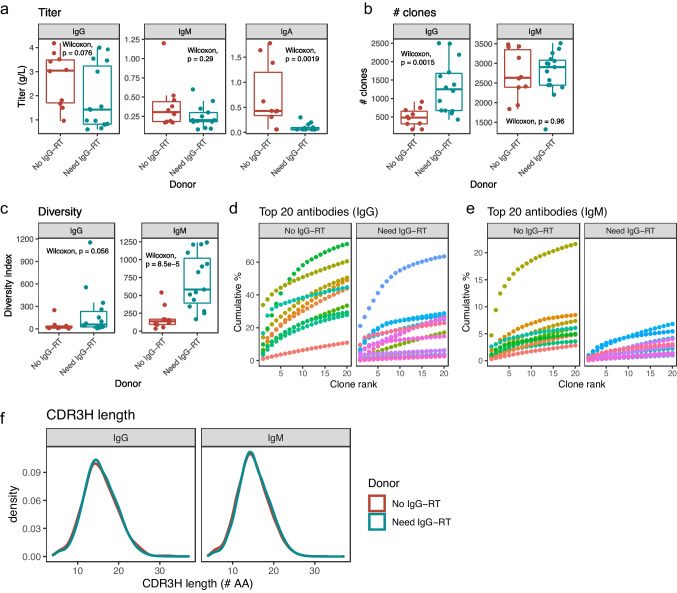
IgG and IgM antibody repertoire sequencing. **a** Antibody titer for IgG (left panel), IgM (middle panel), and IgA (right panel), for the patients who did and did not need IgG-RT. **b** Number of IgG and IgM antibody clones. **c** IgG and IgM antibody diversity indices. **d** Cumulative frequency of the top 20 IgG clones (each patient is a different color). The *y*-axis shows cumulative frequency, measured as percent of the total repertoire, while the *x*-axis shows the top 20 clones, ordered from the most to the least abundant. The right and left panels indicate patients who did and did not need IgG-RT, respectively. **e** Cumulative frequency of the top 20 IgM clones (each patient is a different color). **f** Heavy chain CDR3 amino acid length distribution, for IgG (left panel) and IgM (right panel)

We recruited 25 patients with low IgG serum concentrations of which 15 needed IgG-RT (male, 3; female, 12), and 10 did not need IgG-RT (male, 8; female, 2), based on their susceptibility to infection (Table S1). On average, the patients who needed IgG-RT had 1.86 g/L IgG prior to IgG-RT (standard deviation, SD = 1.31), 0.24 g/L IgM (SD = 0.15), and 0.10 g/L IgA (SD = 0.072) in serum, while the patients who did not need IgG-RT had 2.69 g/L IgG (SD = 1.11), 0.39 g/L IgM (SD = 0.31), and 0.71 g/L IgA (SD = 0.64) in serum (Fig. 1a); the serum IgA titers were significantly different between the two groups (*P* = 0.0019). The patients who needed IgG-RT had a comparable amount of CD19^+^ B cells (mean = 221.3 cells/μL, SD = 146.1) compared to the patients without the need of IgG-RT (208.9 cells/μL, SD = 279.5; *P* = 0.24). Vaccine responses against various pathogens (e.g., tetanus, diphtheria, and pneumococcal polysaccharide) were observed for most patients that did not need IgG-RT compared to patients who did need IgG-RT. Furthermore, autoimmune manifestations, chronic infections, and other complications were more common in patients who needed IgG-RT (Table S1).

### Antibody Repertoire Sequencing

We performed IgG and IgM antibody repertoire sequencing of the heavy chain immunoglobulin for both patient cohorts from isolated PBMCs (IgA repertoires could not be investigated in this study due to low or absent IgA-memory B cell counts in most of the patients in need of IgG-RT; Fig. 1a, Table S1). We defined antibody “clones” conservatively, where unique sequences were combined if they had one amino acid difference within 5–6 amino acid long CDR3H (complementarity-determining region 3 heavy chain), or if they had one to two amino acid differences for >6 amino acid long CDR3H. Only clones with at least two sequencing reads were included in the analysis. Interestingly, the patients who needed IgG-RT had a significantly higher number of IgG clones (mean = 1310 clones) than the patients who did not need IgG-RT (mean = 483 clones; *P* = 0.0015) (Fig. 1b). On the other hand, there was no significant difference in the number of IgM clones between those who did (mean = 2760 clones) and those who did not need IgG-RT (mean = 2733 clones; *P* = 0.96).

To further examine the IgG and IgM antibody repertoires, we measured the true diversity index, which considers the abundance of individual antibody clones in addition to the number of clones. The true diversity of an antibody repertoire X refers to the effective richness of that population: the number of equally common antibody clones that would be required to produce a repertoire with the same overall diversity as X. This value will increase with the number of antibody clones in the repertoire, as well as with the evenness with which these clones are distributed [16]. Relative to those who did not need IgG-RT, the patients who needed IgG-RT had significantly higher IgM diversity index (*P* = 8.5 × 10^−5^) (Fig. 1c).

Among the donors who did not need IgG-RT, one donor had an IgG titer of 4.18 g/L and an additional donor had an IgM titer of 1.2 g/L (Fig. 1a, Table S1). To ensure that these donors with higher antibody titer were not driving the differences in antibody clone counts and diversity, we removed these donors from the datasets (Fig. S1a) and repeated the above analyses. We observed the same differences, where the donors who needed IgG-RT had significantly higher number of IgG clones (*P*= 0.0016; Fig. S1b) and a higher IgM diversity index (*P*= 4.1 × 10^−6^; Fig. S1c).

Visualizations of the frequencies of the top 20 antibody clones showed that patients who needed IgG-RT tended to have less oligoclonal IgG and IgM repertoires (Fig. 1d, e). On average, the top 20 IgG clones made up 19.5% and 42.1% of the total repertoire for the patients who did and the ones who did not need IgG-RT, respectively, indicating lower IgG oligoclonality for the former cohort (*P* = 0.0015). Similarly, the top 20 IgM clones accounted for on average of 2.65% and 7.06% of the repertoire for the patients who did and did not need IgG-RT, respectively, indicating lower IgM oligoclonality for the former cohort (*P* = 0.0014).

We examined the distribution of the CDR3H amino acid sequence lengths, another feature that may provide insight into the composition of the antibody repertoire. However, both patient cohorts had normally distributed heavy chain CDR3 lengths with a median of 15 amino acids, for both IgG and IgM (Fig. 1f).

Together, these data show that the patients who needed IgG-RT had more IgG clones, a higher IgM diversity index, and lower IgG and IgM oligoclonality, consistent with more diverse antibody repertoires.

### Correlations Between Antibody Repertoire and Immune Features

**Fig. 2:**
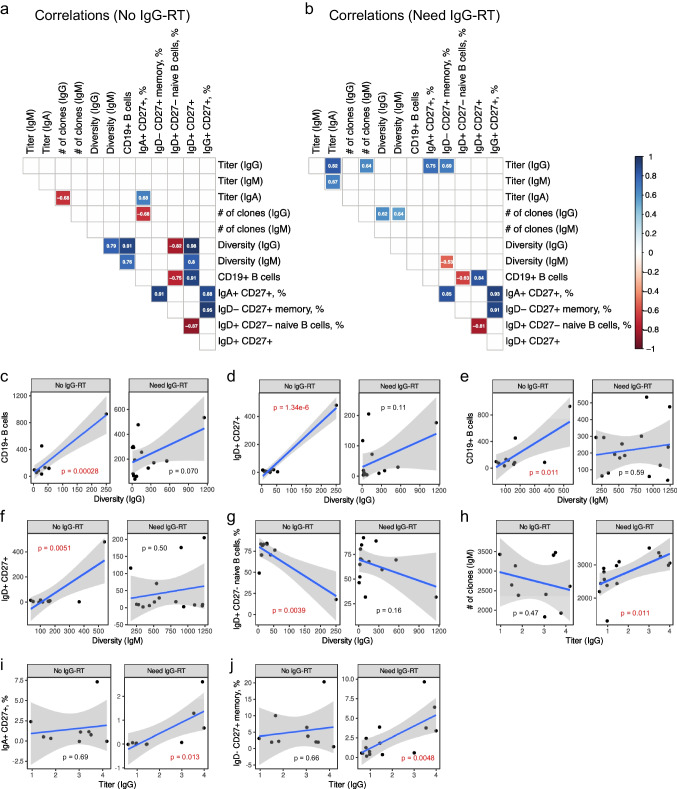
Correlations between antibody repertoire and immune features. **a**, **b** All-by-all correlation matrix of various antibody features and immune cell frequencies for patients who did not (**a**) and did (**b**) need IgG-RT. The numbers indicate Pearson correlation coefficients. Blue and red shadings indicate positive and negative correlation, respectively, as indicated in the legend in **b**. Only significant (*P* ≤ 0.05) correlations are shown. **c**–**j** Scatter plots showing several significant correlations from **a** and **b**, for patients who did (right panels) and did not (left panels) need IgG-RT. The blue lines are linear regression lines while the gray shadings show the 95% confidence intervals around the fitted lines. *P*-values are indicated in black (*P* > 0.05) or red (*P* ≤ 0.05)

Next, we set out to understand the interplay between different features of the antibody repertoires and various immune parameters. For both patient cohorts, we measured the frequency of different B cell subtypes by flow cytometry. Then, we performed an all-by-all correlation analysis of antibody titer, clone count, diversity, and abundance of different B cell subtypes for the patients who did and did not need IgG-RT (Figs. 2, S2; Table S1).

For patients who did not need IgG-RT, IgG diversity positively correlated with the frequency of CD19+ B cells (Pearson correlation coefficient, *r* = 0.91, *P*= 0.00028) and IgD+ CD27+ B cells (*r* = 0.98, *P* = 1.34 × 10^−6^) (Fig. 2a, c, d). In the same patient cohort, IgM diversity also positively correlated with the frequency of CD19+ B cells (*r* = 0.76, *P* = 0.011) and IgD+ CD27+ B cells (*r* = 0.8, *P* = 0.0051) (Fig. 2a, e, f). IgG diversity negatively correlated with the frequency of IgD+ CD27-naïve B cells (*r* =−0.82, *P* = 0.0039) (Fig. 2a, g). These correlations of IgG and IgM diversity with B cell frequencies were not observed in the patients who needed IgG-RT (Fig. 2b).

For patients who needed IgG-RT, the IgG titer correlated with the number of IgM clones (*r* = 0.64, *P* = 0.011) (Fig. 2b, h). The IgG titer also correlated with the frequencies of IgA+ CD27+ B cells (*r* = 0.75, *P* = 0.013) and IgD- CD27+ memory B cells (*r* = 0.69, *P* = 0.0048) (Fig. 2b, i, j). These correlations were not observed in the patients who did not need IgG-RT (Fig. 2a).

### V and J Gene Diversity

**Fig. 3:**
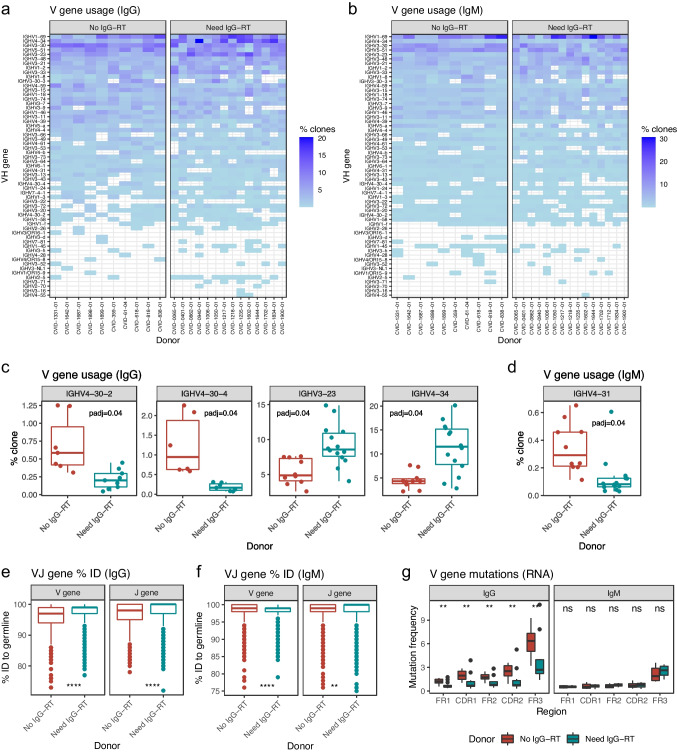
Antibody heavy chain V and J gene diversity. **a** Heatmaps showing the abundance of antibody clones with specific heavy chain V genes (*y*-axis) for the patients (*x*-axis) who did (right panel) and did not need IgG-RT (left panel), for IgG. The color indicates clone frequency per patient, as indicated by the legend. **b** Heatmaps showing heavy chain V gene usage for IgM. **c** IgG heavy chain V genes that are present at different frequencies between the patients who did and did not need IgG-RT. *y*-axis represents percent antibody clone with a given V gene. *P*-values are adjusted using the Benjamini-Hochberg method for multiple testing correction. **d** IgM heavy chain V gene usage difference between patient who did and did not need IgG-RT. **e** Boxplots showing percent nucleotide identity of V and J genes to germline sequences for all IgG clones. **f** Boxplots showing percent amino acid identity of V and J genes to germline sequences for all IgM clones. **g** V gene nucleotide mutation frequency in different regions, for IgG (left panel) and IgM (right panel). FR, framework; CDR, complementarity determining region. ns (not significant): *P* > 0.05, * *P* ≤ 0.05, ** *P* ≤ 0.01, *** *P* ≤ 0.001, **** *P* ≤ 0.0001

V(D)J (variable, diversity, joining) recombination, which assembles antibody gene segments during B cell development, contributes to the vast combinatorial diversity of antibodies [17]. We evaluated whether V(D)J diversity differs between patients who did and did not need IgG-RT. For IgG and IgM, both patient cohorts displayed diverse V and J gene usage (Figs. 3a, b, S3a, S3b). Interestingly, principal component analysis (PCA) of the IgG V gene frequencies revealed that the patients clustered based on their need for IgG-RT. Principal component 1 (PC1), which explained 15.55% of the variance in V gene usage frequencies, separated the patients who did and did not need IgG-RT (Fig. S3c). PCA of IgM V gene usage frequencies showed clustering of the patient cohorts to a lesser extent (Fig. S3d). Next, we compared V gene frequencies between patients who did and did not need IgG-RT. Compared to the patients who did not need IgG-RT, those who needed IgG-RT had fewer IgG antibody clones with the IGHV4-30-2 and IGHV4-30-4 heavy chain V genes (Benjamini-Hochberg adjusted *P*-values = 0.04) (Fig. 3c). The patients who needed IgG-RT also had elevated numbers of antibody clones with the IGHV3-23 and IGHV4-34 V genes (adjusted *P* = 0.04) (Fig. 3c). While the patients who did not need IgG-RT had on average 4.53% of IGHV4-34 clones, consistent with the gene’s 3–9% prevalence in adult B lymphocytes [18], the patients who needed IgG-RT had on average 11.27% of IGHV4-34 antibody clones (Fig. 3c). Notably, antibodies with the IGHV4-34 V gene have been shown to be self-reactive and are more common in naïve B cell repertoire than in memory B cells [19–21]. We also examined differences in IgM V gene usage. The patients who needed IgG-RT had fewer IgM clones with the IGHV4-31 V gene (adjusted *P* = 0.04) (Fig. 3d). Finally, we examined J gene usage frequencies and did not observe any significant difference between the patient cohorts for either IgG or IgM.

Somatic hypermutation, the process in which point mutations accumulate across the antibody V(D)J regions, further contributes to antibody diversity [22]. Somatic hypermutation is also an important means for generating high affinity antibodies. We measured the nucleotide percent identity of the antibody heavy chain V and J gene to their respective germline sequences. Interestingly, compared to patients who did not need IgG-RT, those who needed IgG-RT had significantly higher IgG V and J gene percent germline identity (*P* ≤ 0.0001; Fig. 3e). For IgM, although the V gene percent germline identity was significantly lower for those who needed IgG-RT (*P* ≤ 0.0001; Fig. 3f), the average difference was minor (98.15% for no IgG-RT versus 98.39% for need IgG-RT). The differences in IgG V and J gene percent germline identities remained significant when the donors with the higher IgG/IgM titer were removed from the dataset, suggesting that the observation was not driven by the highest titer donors (Figs. S4a, S4b). To further investigate the difference in V gene mutations between the two patient cohorts, we measured mutation frequencies in different regions along V genes, including the framework regions (FR1, FR2, FR3) and the complementarity determining regions (CDR1, CDR2). The patient cohort who needed IgG-RT had significantly (*P* ≤ 0.05) lower mutation frequencies across all V gene regions, at both the nucleotide level (Figs. 3g, S4c) and the deduced protein level (Fig. S4d, S4e), for IgG but not for IgM. Visualizations of mutation frequencies along the most common V genes further illustrated the lower IgG V gene mutation rates in patients who needed IgG-RT (Figs. S5, S6).

Finally, we measured the frequencies of somatic hypermutation along V gene IGHV4-34 that had elevated usage in IgG for donors who needed IgG-RT. Compared to patients who did not need IgG-RT, patients who needed IgG-RT had lower somatic hypermutations along IGHV4-34 (Fig. S7a). Previous studies indicated that the self-reactivity of IGHV4-34 antibodies is mediated by a hydrophobic patch in the framework 1 region, and that somatic hypermutation in the region can remove self-reactivity [20, 23, 24]. However, there was no significant difference in mutation frequency in the hydrophobic patch (AVY residues) when comparing the two cohorts (Fig. S7b).

Overall, these data show that IgG hypogammaglobulinemia patients who did and did not need IgG-RT had antibody repertoires with different V gene diversities. Patients who needed IgG-RT displayed higher usage of a naïve antibody repertoire-associated V gene and had less somatic hypermutation in their IgG clones, possibly suggesting less mature antibody repertoires leaving these patients more susceptible to infection.

## Discussion

The decision to treat hypogammaglobinemia patients with IgG-RT can be challenging, because both IgG levels and infection susceptibility vary among patients. IgG levels do not always predict a patient’s infection susceptibility, and in some cases, IgG-RT is recommended for patients with asymptomatic hypogammaglobulinemia because of the potential risk of severe infections [25]. Furthermore, both symptomatic and asymptomatic hypogammaglobinemia patients can respond well to tetanus vaccines, while diphtheria response is often impaired. Indeed, most patients in this study had a positive response to tetanus vaccine, before IgG-RT started for those who need it, while many did not respond to diphtheria (Table S1). In addition, 8 of 9 patients who did not need IgG-RT that were vaccinated with pneumococcal polysaccharides had a positive response, while only 1 of 4 patients who needed IgG-RT responded.

Hypogammaglobulinemia patients who did and did not need IgG-RT had multiple differences in their peripheral B cell receptor repertoires. Patients who needed IgG-RT had more IgG antibody clones, a higher IgM diversity index, and less oligoclonal IgG and IgM repertoires. Their IgG clones displayed distinct heavy chain V gene usage, had higher frequencies of sequences with a naïve B cell repertoire-associated V gene, and their IgG clones had less somatic hypermutation and looked more similar to germline sequences. The lower level of clonal antibody expansion and somatic hypermutation suggests that these infection susceptible patients have relatively immature B cell receptor repertoires that may be less effective against pathogens. A reduced frequency of somatic hypermutation was found in the B cell receptor repertoire of common variable immunodeficiency (CVID) patients as well, further suggesting impaired repertoire specification in the germinal centers [26, 27]. Interestingly, the patients in need of IgG-RT showed increased IGHV4-34 and IGHV3-23 gene usage compared to the patients without the need of IgG-RT. The IGHV4-34 increase was observed in CD19-deficient patients, patients with Wiskott–Aldrich syndrome (WAS), and RAG deficiency patients, indicating its role in self-reactive autoantibodies. Tipton et al. summarized reports of increased IGHV4-34 gene usage in systemic lupus erythematosus patients, concluding another hallmark in the repertoire of the disease, defective tolerance, and 9G4-idiotype autoantibodies [28]. The IGHV3-23 gene has been shown to be associated with the exposure to self and/or environmental antigens [29] and is relatively abundant in humans [30, 31]. IGHV3-23 gene usage was also reported in hairy cell leukemia [32], diffuse large B-cell lymphoma [33], after the immunization of malaria-naïve individuals with PfSPZ-CVac [34], HIV patients [35], and in CD21(low) B cells from WAS patients [36].

Conversely, hypogammaglobulinemia patients who did not need IgG-RT had relatively expanded and antigen-experienced B cell repertoires that appear to be adapted to better overcome infection susceptibility. These patients revealed elevated gene usage of IGHV4-30-2, IGHV4-30-4, and IGHV4-31 compared to the patients in need of IgG-RT. An increase of IGHV4-30-2 and -4 has been reported in WAS patients as well, demonstrating abnormalities of immune repertoire in both cohorts [37]. Another study on plasmablasts from patients with multiple sclerosis revealed a positive and negative employment of IGHV4-30 gene usage, suggesting other factors influencing autoreactive property, such as CDR3 length and charge, light chain pairing, or mutation accumulation [38]. Two patients with primary cutaneous follicle center lymphoma (FCL) showed increase usage of the IGVH4-30 gene, indicating the relevance of pathological antigen epitopes in cutaneous lymphomagenesis [39]. Naïve and memory B cells from WAS patients showed increased IGVH4-31 gene usage [36], observed in our patients with no need for IgG-RT. The Simon et al. study reported an age-dependent deterioration of B-cell differentiation possibly leading to an increased infection susceptibility and autoimmune manifestations. Similar observations have been reported in nodal marginal zone lymphomas [40] and cerebrospinal fluid B cells of patients with multiple sclerosis [41, 42]. Furthermore, the synovium of patients with rheumatoid arthritis both with and without anti-glucose-6-phosphate isomerase antibodies showed frequent IGVH4-31 gene usage [43]. And interestingly, the two patients in this study with possible secondary hypogammaglobulinemia who did not need IgG-RT showed no differences compared to the primary hypogammaglobulinemia patients that did not need IgG-RT.

An important caveat to our study is that we profiled the antibody receptor repertoires derived from peripheral B cells that represented only a snapshot of the adaptive B cell response from the time of blood collection. Future studies should include the analysis of the repertoire profiles from bone marrow-derived plasma B cells and across longitudinal time points, which may provide a more comprehensive analysis of the adaptive immune response in these patients. Furthermore, because IgG-RT has direct and indirect effects on B cell development, by binding to surface receptors or intracellular molecules and by the influence of cytokines, survival factors, and other immune cells [44], it is possible that the IgG-RT treatment itself could be responsible for some of the B cell repertoire changes observed between the two patient cohorts. Nevertheless, our study shows that peripheral B cell receptor sequencing may be utilized in the decision-making process for or against the use of IgG-RT in the setting of hypogammaglobulinemia.

## Supplementary Information

ESM 1

ESM 2

ESM 3

## Data Availability

The sequencing datasets generated for the current study are available in the SRA repository, BioProject: PRJNA876301.
