# Supplementary material for: Sequencing the B Cell Receptor Repertoires of Antibody-Deficient Individuals With and Without Infection Susceptibility
Source: J Clin Immunol. 2023 Feb 24;43(5):940–50. doi: 10.1007/s10875-023-01448-0 (PMC10276080; doi:10.1007/s10875-023-01448-0)
Supplement: Supplementary file 3 — ESM 3 [file 10875_2023_1448_MOESM3_ESM.pptx]

## Slide 1
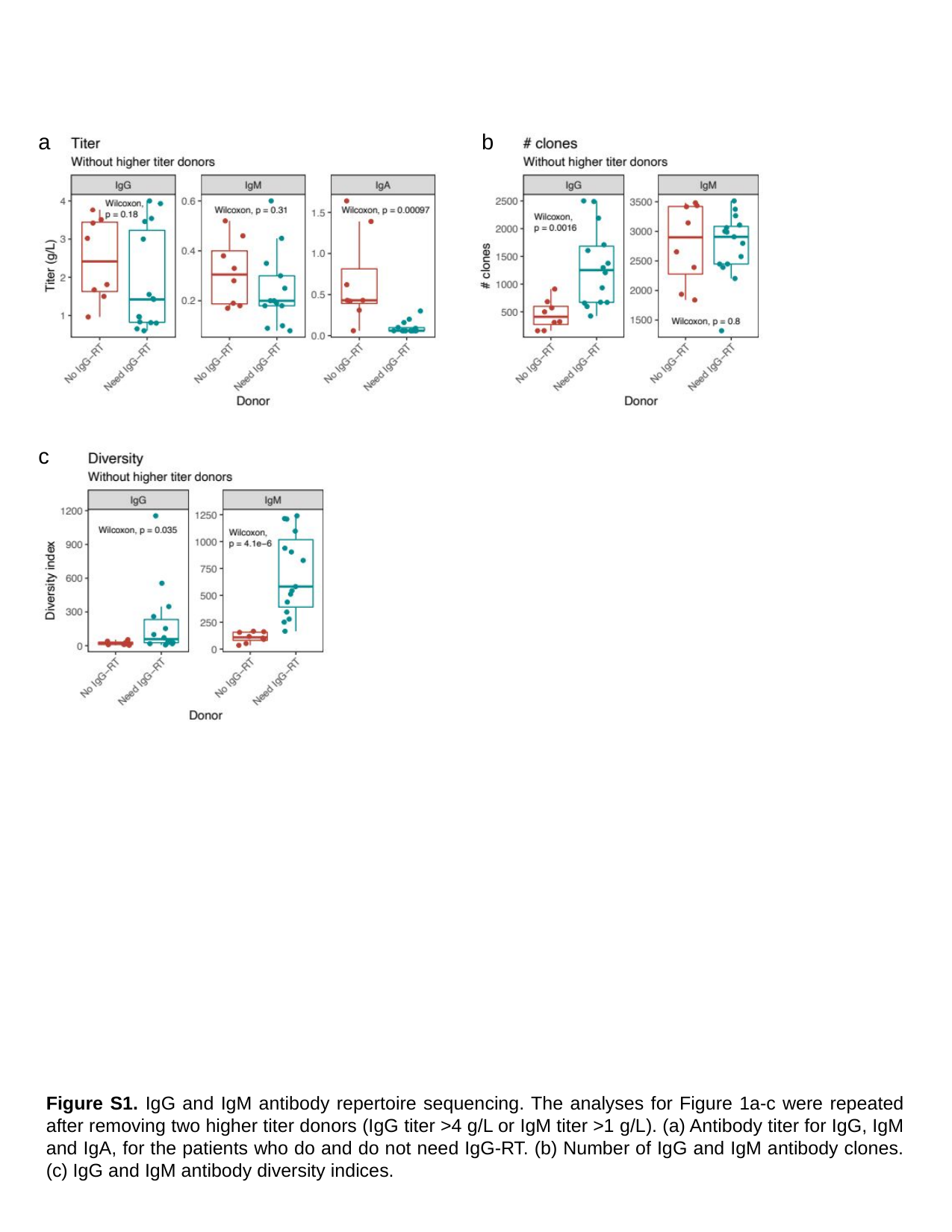

a
b
c
Figure S1. IgG and IgM antibody repertoire sequencing. The analyses for Figure 1a-c were repeated after removing two higher titer donors (IgG titer >4 g/L or IgM titer >1 g/L). (a) Antibody titer for IgG, IgM and IgA, for the patients who do and do not need IgG-RT. (b) Number of IgG and IgM antibody clones. (c) IgG and IgM antibody diversity indices.

## Slide 2
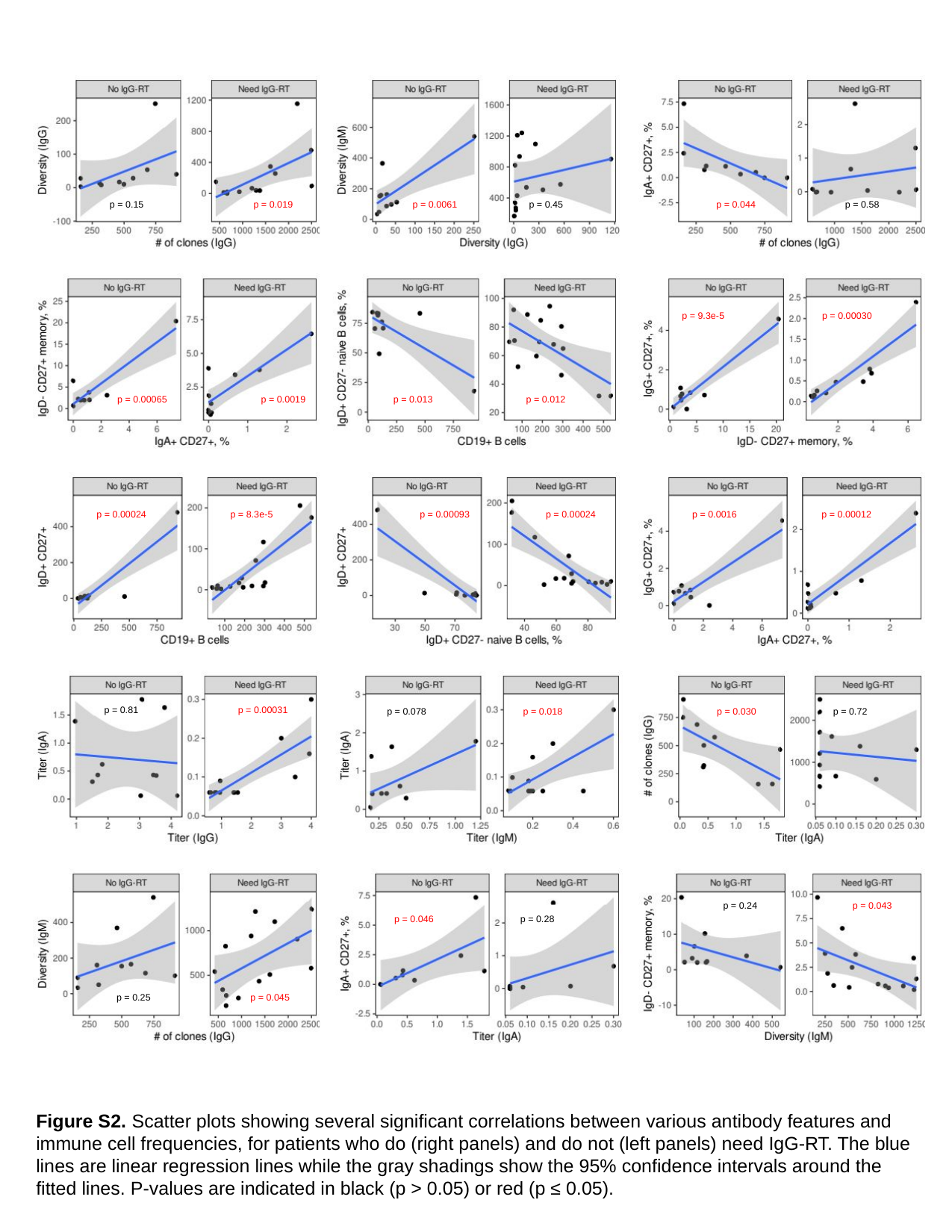

p = 0.15
p = 0.019
p = 0.0061
p = 0.45
p = 0.044
p = 0.58
p = 0.00065
p = 0.0019
p = 0.013
p = 0.012
p = 9.3e-5
p = 0.00030
p = 0.00024
p = 8.3e-5
p = 0.00093
p = 0.00024
p = 0.0016
p = 0.00012
p = 0.81
p = 0.00031
p = 0.078
p = 0.018
p = 0.030
p = 0.72
p = 0.24
p = 0.043
p = 0.046
p = 0.28
p = 0.25
p = 0.045
Figure S2. Scatter plots showing several significant correlations between various antibody features and immune cell frequencies, for patients who do (right panels) and do not (left panels) need IgG-RT. The blue lines are linear regression lines while the gray shadings show the 95% confidence intervals around the fitted lines. P-values are indicated in black (p > 0.05) or red (p ≤ 0.05).

## Slide 3
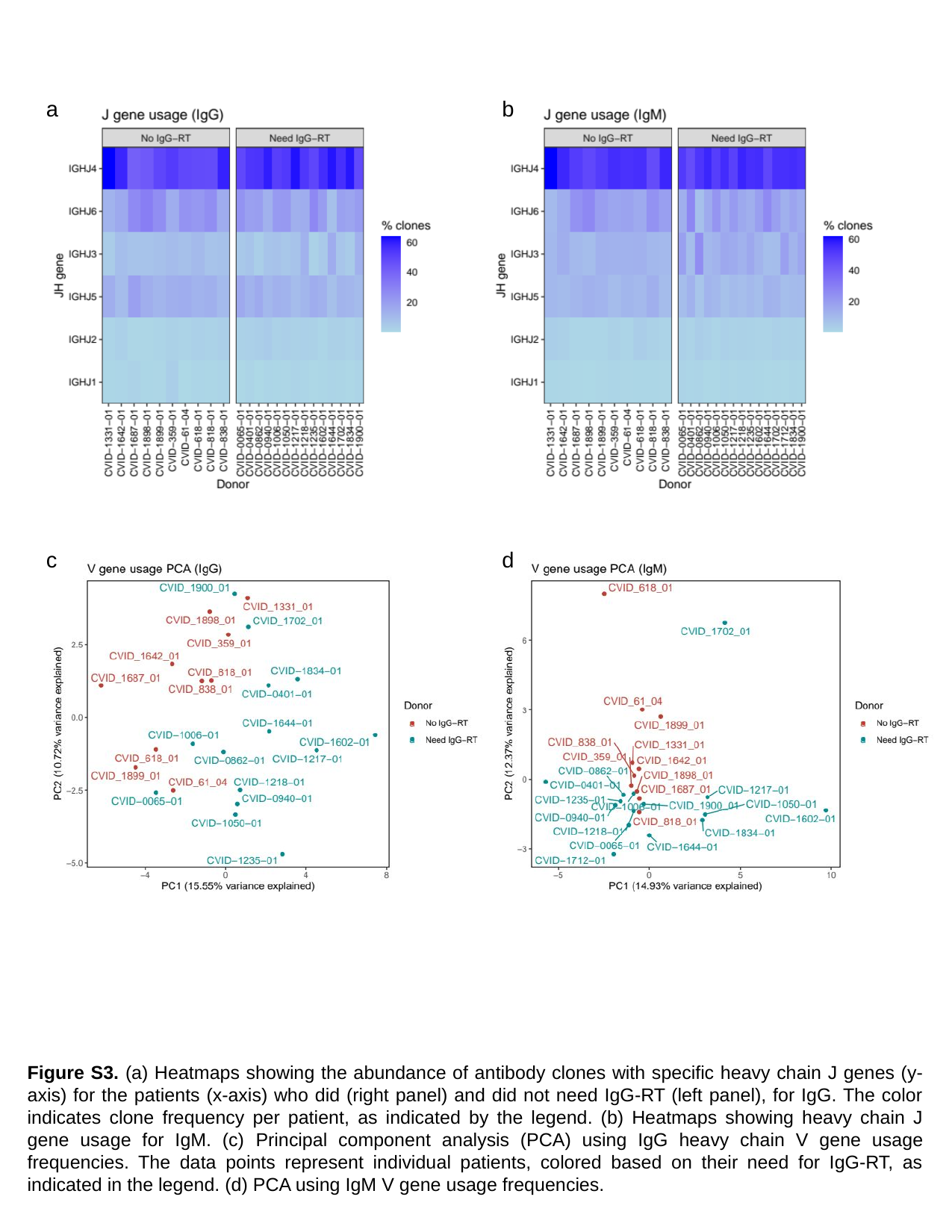

a
b
c
d
Figure S3. (a) Heatmaps showing the abundance of antibody clones with specific heavy chain J genes (y-axis) for the patients (x-axis) who did (right panel) and did not need IgG-RT (left panel), for IgG. The color indicates clone frequency per patient, as indicated by the legend. (b) Heatmaps showing heavy chain J gene usage for IgM. (c) Principal component analysis (PCA) using IgG heavy chain V gene usage frequencies. The data points represent individual patients, colored based on their need for IgG-RT, as indicated in the legend. (d) PCA using IgM V gene usage frequencies.

## Slide 4
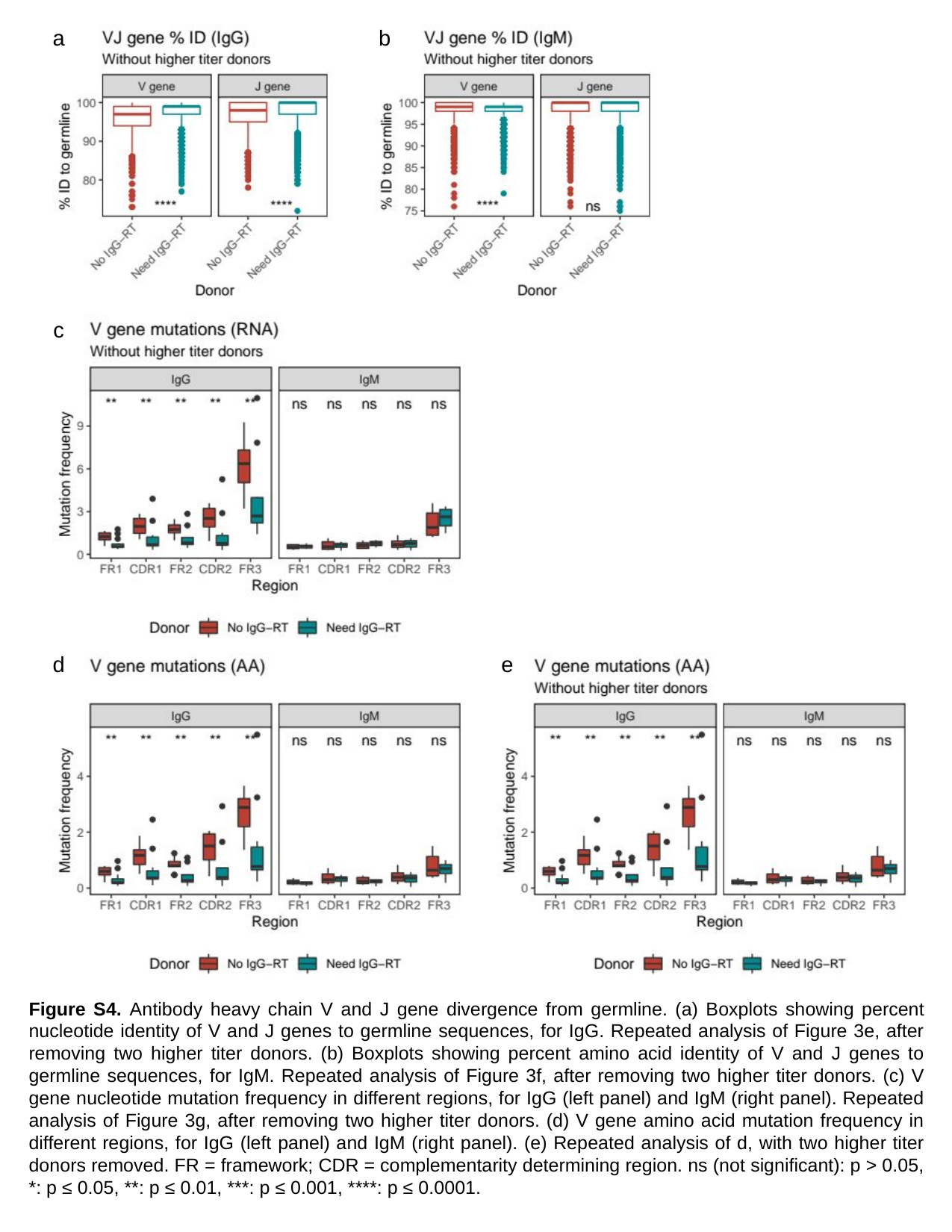

a
b
c
d
e
Figure S4. Antibody heavy chain V and J gene divergence from germline. (a) Boxplots showing percent nucleotide identity of V and J genes to germline sequences, for IgG. Repeated analysis of Figure 3e, after removing two higher titer donors. (b) Boxplots showing percent amino acid identity of V and J genes to germline sequences, for IgM. Repeated analysis of Figure 3f, after removing two higher titer donors. (c) V gene nucleotide mutation frequency in different regions, for IgG (left panel) and IgM (right panel). Repeated analysis of Figure 3g, after removing two higher titer donors. (d) V gene amino acid mutation frequency in different regions, for IgG (left panel) and IgM (right panel). (e) Repeated analysis of d, with two higher titer donors removed. FR = framework; CDR = complementarity determining region. ns (not significant): p > 0.05, *: p ≤ 0.05, **: p ≤ 0.01, ***: p ≤ 0.001, ****: p ≤ 0.0001.

## Slide 5
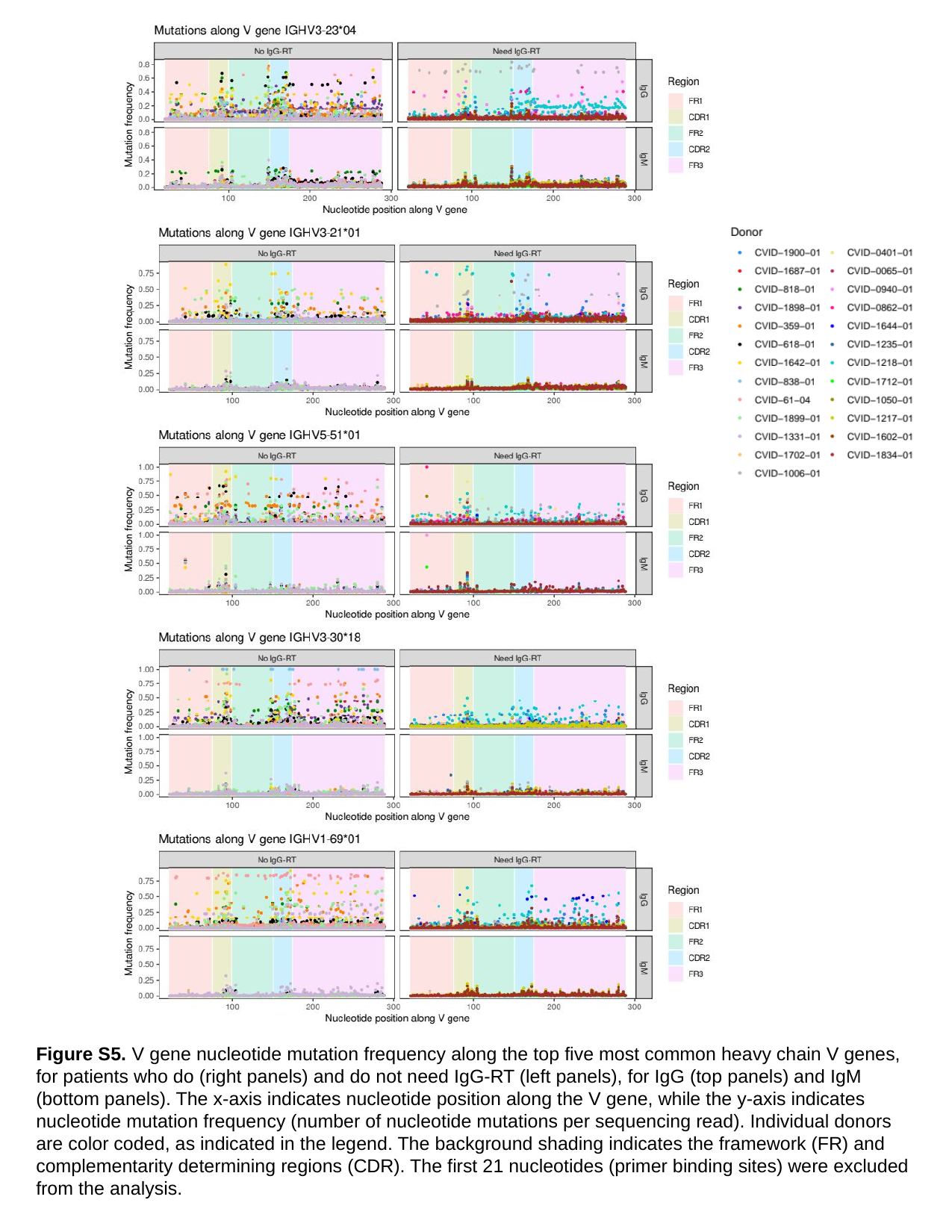

Figure S5. V gene nucleotide mutation frequency along the top five most common heavy chain V genes, for patients who do (right panels) and do not need IgG-RT (left panels), for IgG (top panels) and IgM (bottom panels). The x-axis indicates nucleotide position along the V gene, while the y-axis indicates nucleotide mutation frequency (number of nucleotide mutations per sequencing read). Individual donors are color coded, as indicated in the legend. The background shading indicates the framework (FR) and complementarity determining regions (CDR). The first 21 nucleotides (primer binding sites) were excluded from the analysis.

## Slide 6
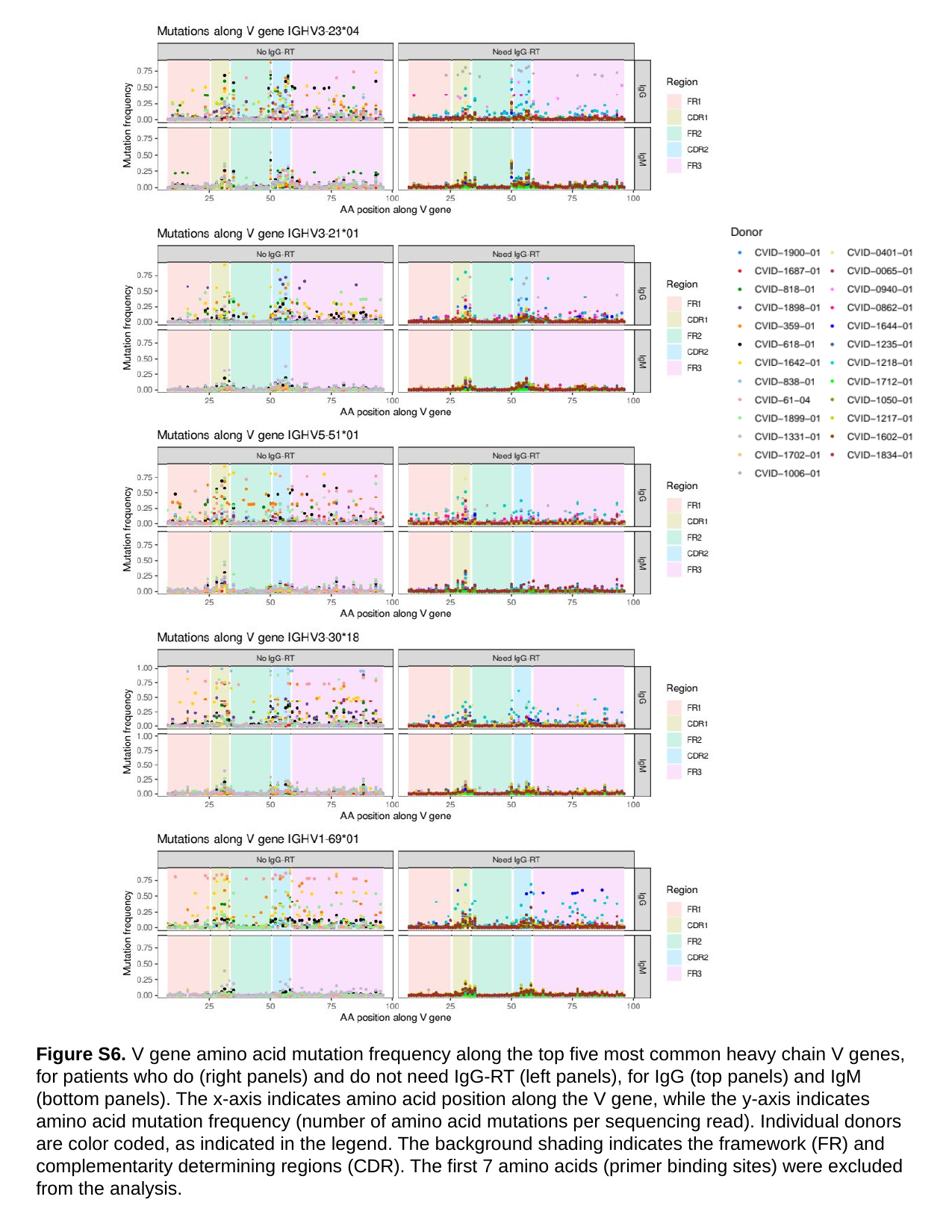

Figure S6. V gene amino acid mutation frequency along the top five most common heavy chain V genes, for patients who do (right panels) and do not need IgG-RT (left panels), for IgG (top panels) and IgM (bottom panels). The x-axis indicates amino acid position along the V gene, while the y-axis indicates amino acid mutation frequency (number of amino acid mutations per sequencing read). Individual donors are color coded, as indicated in the legend. The background shading indicates the framework (FR) and complementarity determining regions (CDR). The first 7 amino acids (primer binding sites) were excluded from the analysis.

## Slide 7
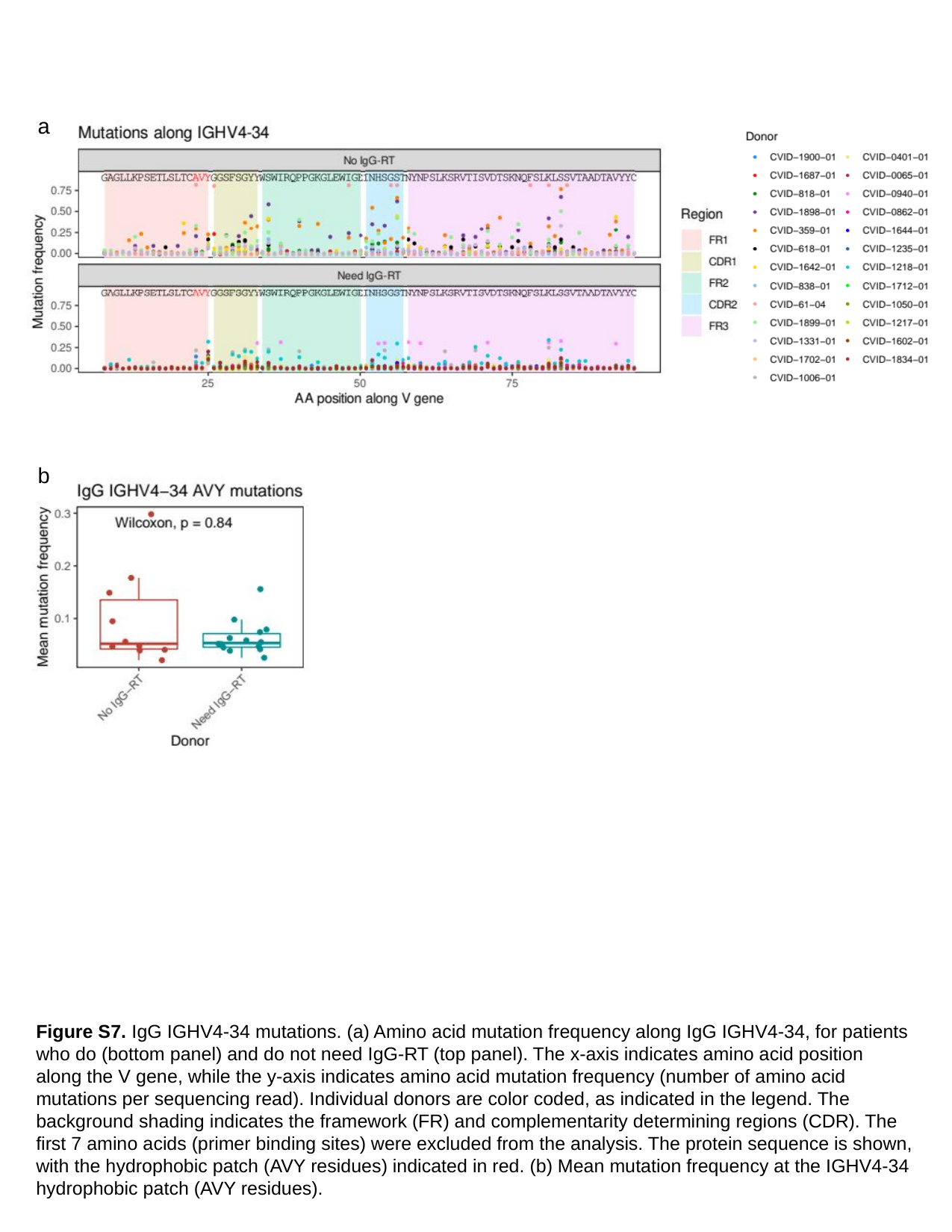

a
b
Figure S7. IgG IGHV4-34 mutations. (a) Amino acid mutation frequency along IgG IGHV4-34, for patients who do (bottom panel) and do not need IgG-RT (top panel). The x-axis indicates amino acid position along the V gene, while the y-axis indicates amino acid mutation frequency (number of amino acid mutations per sequencing read). Individual donors are color coded, as indicated in the legend. The background shading indicates the framework (FR) and complementarity determining regions (CDR). The first 7 amino acids (primer binding sites) were excluded from the analysis. The protein sequence is shown, with the hydrophobic patch (AVY residues) indicated in red. (b) Mean mutation frequency at the IGHV4-34 hydrophobic patch (AVY residues).
